# Simple Synthesis and Characterization of Shell-Thickness-Controlled Ni/Ni_3_C Core-Shell Nanoparticles

**DOI:** 10.3390/nano12121954

**Published:** 2022-06-07

**Authors:** Sun-Woo Kim, Jae Chul Ro, Su-Jeong Suh

**Affiliations:** The School of Advanced Materials Science and Engineering, SungKyunKwan University, Seobu-ro, Jangan-gu, Suwon-si 2066, Gyeonggi-do, Korea; swz001@skku.edu (S.-W.K.); rojc33@skku.edu (J.C.R.)

**Keywords:** nickel carbide, carburization, core-shell structure, magnetic materials

## Abstract

Ni/Ni_3_C core-shell nanoparticles with an average diameter of approximately 120 nm were carburized via a chemical solution method using triethylene glycol. It was found that over time, the nanoparticles were covered with a thin Ni_3_C shell measuring approximately 1–4 nm, and each Ni core was composed of poly grains. The saturation magnetization of the core-shell nanopowders decreased in proportion to the amount of Ni_3_C. The synthesis mechanism of the Ni/Ni_3_C core-shell nanoparticles was proposed through X-ray diffraction (XRD), X-ray photoelectron spectroscopy (XPS) and transmission electron microscopy (TEM) analyses.

## 1. Introduction

Nanosized magnetic materials of Fe, Co, and Ni have received much attention owing to their unique magnetic, catalytic, and optical properties, as well as their promising applications in magnetic sensors, high-density magnetic recording, catalysis, and biomedicine [1,2,3,4,5,6,7]. Nickel carbide (Ni_3_C) has received increasing attention because of its remarkable chemical stability, catalytic properties, and magnetic properties. Nanocomposite magnetic materials with a combination of two nanoscale phases have attracted significant attention because of their ability to generate magnetic properties that are not achievable by a single material [8,9]. Typically, Ni_3_C does not exist in nature owing to its instability. It is mainly obtained by physical methods under a high temperature and pressure, such as C-ion implantation into Ni, mechanical alloying, and the reaction of Ni with an amorphous C film [10,11,12,13,14]. Recently, a mild chemical solution method was employed in the synthesis of nanosized Ni_3_C. Most core-shell Ni-based nanomaterials have been reported to be Ni/C or Ni_3_C/C nanoparticles [15,16,17,18,19]. In previous studies, the synthesis was performed by the chemical solution method, and the shell structure of Ni3C was obtained at a low temperature. Although this experiment was also a chemical method, a shell structure of nickel carbide was obtained at a relatively low temperature. Ni/Ni_3_C core-shell nanoparticles (NICs) were synthesized at a lower temperature (523 K) by a simple two-step chemical solution method involving the synthesis of Ni nanoparticles (Ni Nps) and carburization, following which the structure, morphology, and related magnetic properties of the nanomaterials were studied. The structure, shape, and related magnetic properties of the nanomaterials were analyzed using the synthesized materials, and their growth mechanisms were studied. In addition, because of the non-ferromagnetism of Ni_3_C and ferromagnetism of Ni, the effect of carburization on the magnetic properties of Ni was investigated.

## 2. Materials and Methods

### 2.1. Synthesis of Ni Nps

(1) 0.5 g of a nickel chloride hexahydrate (NiCl_2_. 6H_2_O, 98%, Daejung, Siheung, Korea) precursor was dissolved in 60 mL of deionized water. (2) 1 g of sodium hydroxide (NaOH, 97%, Junsei, Tokyo, Japan) was added to 20 mL of deionized water. (3) 20 mL of hydrazine monohydrate (H_4_N_2_·H_2_O, 99%, Daejung, Siheung, Korea) was prepared as a reducing agent. After mixing them all, the solution with a light green color (arising from the Ni ions) turned blue. After heating the solution to 60 °C, ultrasonic treatment for 25 min resulted in the formation of black Ni Nps. These Nps were collected and washed several times with acetone and deionized water. After washing, the samples were stored in acetone.

### 2.2. Carburization to Obtain Ni/Ni_3_C Core-Shell Nanoparticles (NICs)

The prepared Ni Nps were transferred to a triethylene glycol (TEG, C_6_H_14_O_4_, 97%, Daejung, Siheung, Korea) solution that contained a small amount of NaOH. NICs were obtained by heating the solution to 250 °C at a heating rate of 8 °C/min for 15, 30, 45, and 60 min (resulting samples denoted as NIC15, NIC30, NIC45, and NIC60, respectively) at a rotational speed of 250 rpm.

### 2.3. Analysis of Samples

The phase compositions of the Ni Nps and NICs were identified by X-ray diffraction (XRD; D8 Advance HP, Bruker, Billerica, MA, USA) with Cu Kα radiation at 40 kV and 150 mA. The patterns were recorded over an angular range of 10–90° (2θ) at a scan rate of 5 °/min. The shape, size, and crystallinity of the samples were analyzed using scanning electron microscopy (SEM; XL30 FEG, Philips, Amsterdam, Netherlands) and high-resolution transmission electron microscopy (HRTEM; JEM F 200, JEOL, Tokyo, Japan). Binding energy observations to confirm the increase in the C content were performed using X-ray photoelectron spectroscopy (XPS ESCA2000, VG Microtech, London, UK). The magnetic properties of the prepared samples were measured using a vibrating sample magnetometer (VSM; DMS1660, Microsense, Lowell, MA, USA).

## 3. Results and Discussion

**Figure 1:**
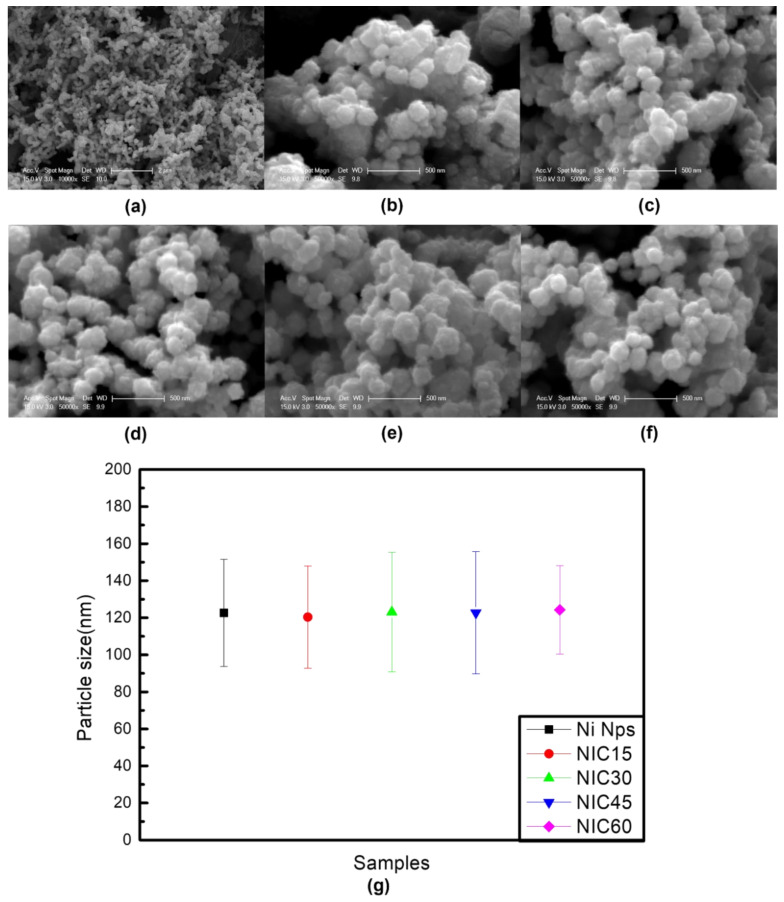
(**a**,**b**) SEM images of the Ni Nps at different magnifications. (**c**–**f**) SEM images of NICs, which were prepared through time-based carburization (15, 30, 45, and 60 min). (**g**) Graph of the mean particle size with deviations for the Ni Nps and NICs.

Figure 1 shows the SEM images of the Ni Nps and NICs. Figure 1a,b show images at different magnifications, indicating that the Ni Nps are formed in a slightly distorted spherical shape. In Figure 1c–f, for each synthesis time, the NICs do not appear to differ significantly from the Ni Nps. As summarized in Figure 1g, the average size of the Ni Nps is 122.64 nm and those of the NICs are 120.33, 123.08, 122.67, and 124.29 nm, with respect to the processing time. It was difficult to observe changes in the shape or size of the NICs due to the reaction and temperature during carburization.

**Figure 2:**
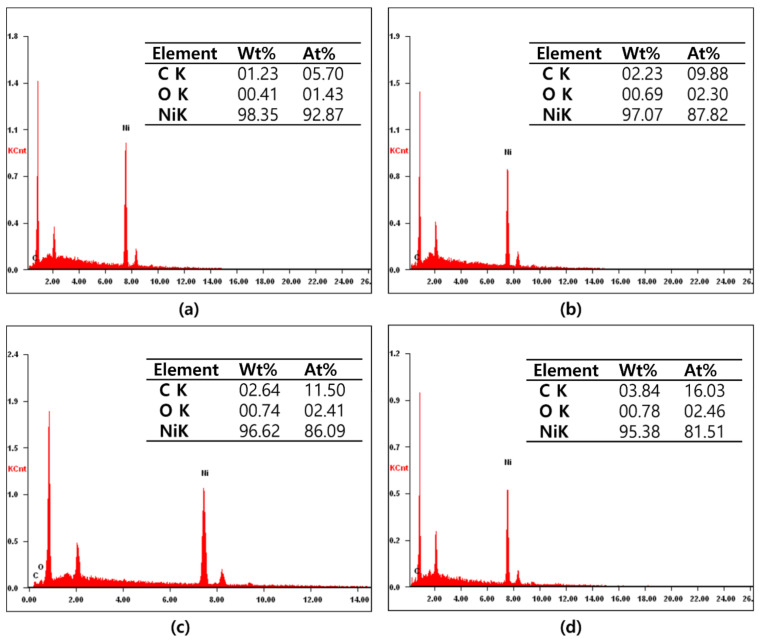
(**a**–**d**) EDS data of NICs prepared using TEG at carburizing process times of 15, 30, 45, and 60 min.

Figure 2a–d show the EDS data of the NICs synthesized by carburization according to the process time (15, 30, 45, and 60 min). With an increasing processing time of the NICs, the atomic percentage of Ni gradually decreases, but those of C increase to 5.7, 9.88, 11.5, and 16.03%. Thus, it was determined that C was gradually added to the Ni Nps according to the processing time.

**Figure 3:**
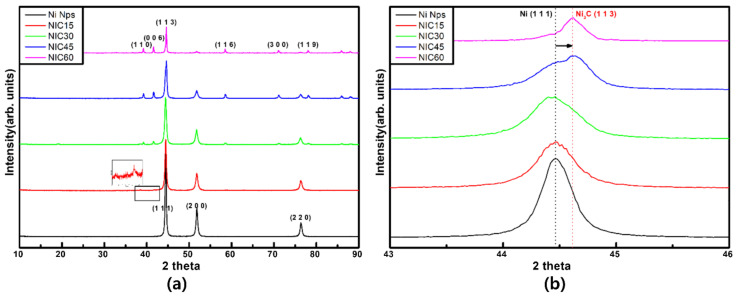
(**a**) XRD data of Ni Nps and NICs. (**b**) Enlarged graph showing the major peaks of Ni and Ni_3_C.

Figure 3a shows the XRD patterns of the Ni Nps and NICs prepared at various processing times. All samples exhibit a single phase without impurities. The Ni Nps exhibit peaks at 2θ values of 44.48, 51.83, and 76.35°, corresponding to the (111), (200), and (220) crystal planes of Ni (JCPDS card number 70-1849). As the carburization time increases, the peak corresponding to Ni decreases and gradually peaks at positions 39.27, 41.75, 44.52, 58.62, 71.20, and 78.10°, corresponding to the (110), (006), (113), (116), (300), and (119) planes and indexed with Ni_3_C (JCPDS card number 06-0697) [19]. Figure 3b is an enlarged graph of the Ni(111) and Ni_3_C(113) peaks, which are the major diffraction peaks of Ni and Ni_3_C. A peak shift occurs as the processing time increases. This is because the C atom occupies an interstitial position, resulting in a shift of the peak toward a higher angle [20,21,22].

**Figure 4:**
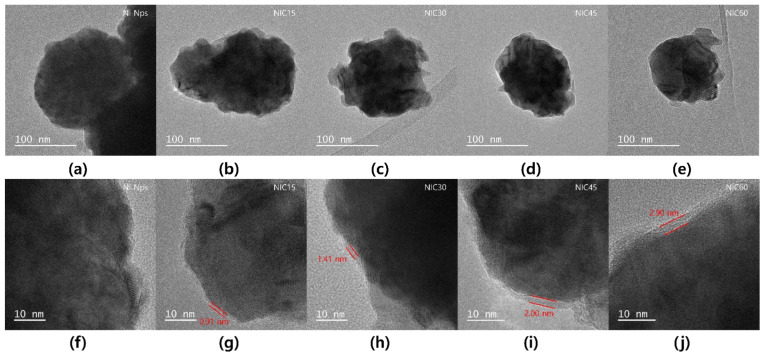
TEM images of (**a**,**f**) Ni Nps, (**b**,**g**) NIC15, (**c**,**h**) NIC30, (**d**,**i**) NIC45, and (**e**,**j**) NIC60.

Figure 4 is a time-dependent TEM image of the sample before and after carburization. In Figure 4a–e, the same coarse spherical particles as observed in the SEM images are noticeable. In Figure 4f–j, which are the high-magnification bright-field TEM images, the Ni_3_C shell layer on the particle surface gradually increases in size to 0.91, 1.41, 2.00, and 2.90 nm with an increasing treatment time. In other words, this result shows that the thickness of the Ni_3_C shell on the Ni core can be tuned with the processing time.

**Figure 5:**
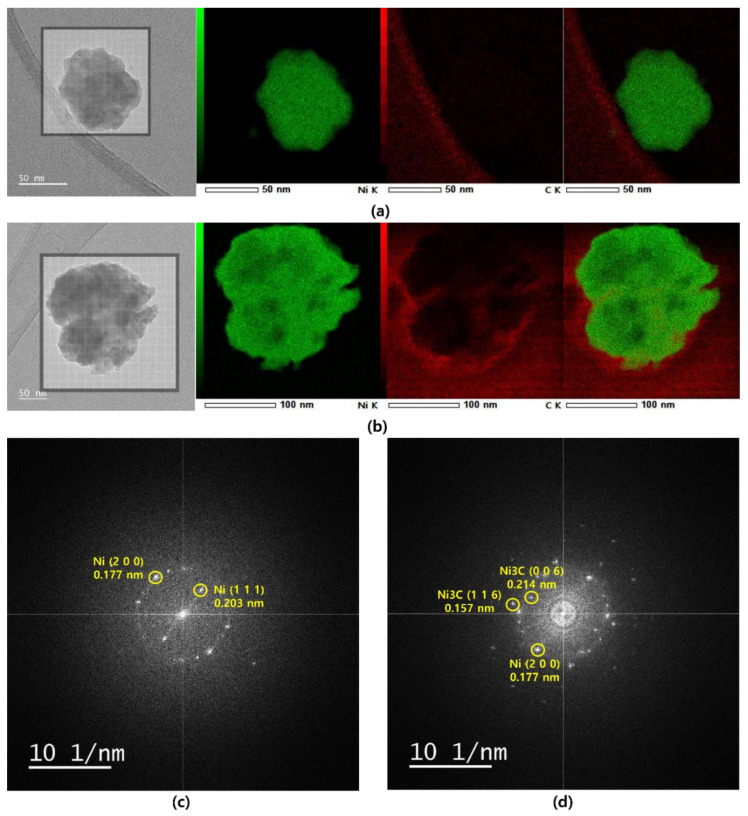
TEM images and corresponding EDS elemental maps of (**a**) Ni Nps and (**b**) NIC60, revealing the spatial distribution of Ni and C elements. (**c**) Fast Fourier transform (FFT) of Ni Nps. (**d**) Fast Fourier transform (FFT) of NIC60.

Figure 5 shows the EDS elemental maps of the Ni Nps and NIC60 captured from the enclosed regions in the TEM images for Ni and C. In Figure 5a, there is no C element around Ni except for the C-supported TEM grid. However, in Figure 5b, a large amount of C is present in the Ni shell. This confirms that C is carburized on the surface of Ni through the employed process. The fast Fourier transform (FFT) pattern of the Ni Nps is shown in Figure 5c. The diffraction pattern of the Ni Nps is ring-like and shows polycrystalline characteristics. The indicated diffraction spots in the FFT pattern correspond to each grating region. From the inverse calculation of the D spacing through the location of the spot with a high intensity, the plane spacings are 0.204 and 0.177 nm. This implies that 0.204 nm and 0.177 nm are the D-spacings in the (111) and (200) plane directions of Ni, respectively. Hence, the Ni Nps appear to be pure Ni polycrystals. The fast Fourier transform (FFT) pattern of NIC60 is also shown in Figure 5d. The diffraction pattern in the proximity of the NIC60 shell shows ring-like polycrystalline characteristics. From the inverse calculation of the D spacing through the location of the spot with a high intensity, the plane spacings are determined to be 0.214, 0.157, and 0.177 nm. Here, 0.214 and 0.157 nm are the D spacings in the (006) and (116) plane directions of Ni_3_C, respectively, and 0.177 nm is the D spacing in the (200) plane direction of Ni. This confirms that NIC60 has a nickel–nickel carbide core-shell structure.

**Figure 6:**
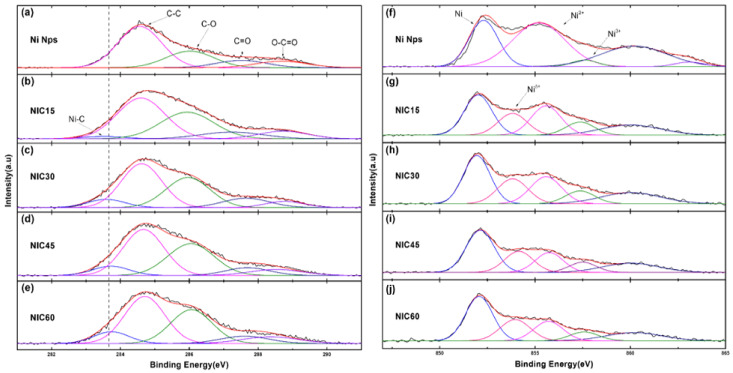
XPS C1s data of (**a**) Ni Nps, (**b**) NIC15, (**c**) NIC30, (**d**) NIC45, and (**e**) NIC60. XPS Ni2P_2/3_ data of (**f**) Ni Nps, (**g**) NIC15, (**h**) NIC30, (**i**) NIC45, and (**j**) NIC60.

The XPS C1s spectra (Figure 6a–e) were measured to study the elemental composition and bonding states, and to confirm that carbon did not exist separately on the surface but reacted with Ni. A broad peak was observed in the C1s range of 281.0–290.0 eV, and five distinct binding energy (BE) peaks were obtained by deconvolution, assignable to five C species. The observed C1s peak at 284.7 eV is consistent with the C1s peak of the C-C bond. The C1s peak BEs of 286.1, 287.2, and 288.7 eV correspond to the C-O, C=O, and O-C=O bonds, respectively [23,24]. Unlike the Ni Nps, the NICs subjected to carburization exhibit a BE of 283.5 eV [25,26]. This can be attributed to the C1 of the Ni-C bond. The peak for the BE of 283.5 eV tends to increase with the processing time, which implies that more reactions occur and more C enters the Ni Nps.

The oxidation state of nickel in the surface region can be determined from the BE and chemical shift in the XPS Ni 2P_2/3_ spectrum (Figure 6f–j). Since nickel naturally oxidizes, such oxidation-related peaks appear for Ni Nps. The results of previous studies support the assignment of BEs of 852.6, 855–856, and 857–858 eV in the Ni 2P_3/2_ XPS spectra for Ni0, Ni^2+^, and Ni^3+^ in oxidized Ni, oxides, and hydroxides and oxyhydroxides, respectively [27,28,29]. The BE of 860.2 eV is characteristic of related satellite peaks. The slightly lower BE of 854.5 eV is assigned to partially reduced nickel species with a positive charge δ+ (Ni^δ+^), indicative of the presence of a nickel carbide phase [30,31,32,33]. As the synthesis time increases, the ratio of this peak to the 2+ peak gradually increases, indicating that the ratio of nickel carbide in the powder increases.

**Figure 7:**
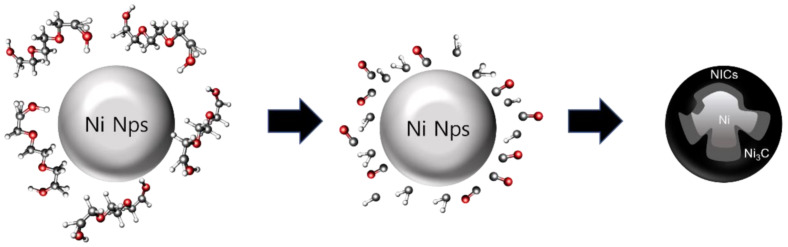
Suggested growth mechanism of NICs.

The formation of the Ni/Ni_3_C core-shell structure can be described using the following simplified mechanism: TEG molecules derived from electrostatic interactions are adsorbed onto the polycrystalline Ni Nps surface. Activated C and CO are formed by the thermal decomposition of ethylene glycol [34] on the surface of the Ni particles. Subsequently, the activated C atoms and CO gradually diffuse from the surface to the Ni Nps by the C concentration gradient to form Ni3C compounds through the highly reactive polycrystalline grain boundaries and the catalytic activity of the Ni particles themselves [14]. Thus, an intact Ni core is formed in the center, and a core-shell structure is finally achieved. In summary, through the mechanism shown in Figure 7, it is inferred that carbon is activated through the thermal decomposition of TEG at a low temperature (less than 523 K) and the diffusion of Ni_3_C due to the catalytic role of polycrystalline Ni Nps that contribute to the formation of NICs. This is similar to the results of previous studies on cobalt carbide [35].

**Figure 8:**
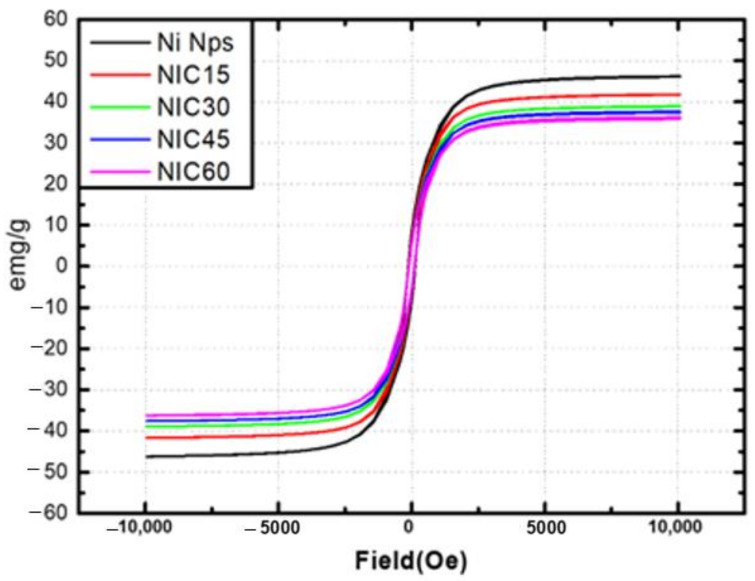
Field-dependent magnetization (M−(H) curves) of Ni Nps and NICs at 300 K.

Figure 8 shows the magnetic hysteresis loops (M−H curves) at room temperature for the Ni Nps and NICs. A hysteresis loop was recorded after applying a magnetic field of up to 10,000 Oe. The saturation magnetization (Ms) of bulk Ni is 55.4 emu/g, and that of the Ni Nps is reduced to 46.2 emu/g. The number of spins on the surface increases as the particle size decreases; thus, the spin canting effect increases. Therefore, the Ms of the nanoparticles is lower than that of the bulk [36,37,38]. As shown, the Ms values of the samples obtained at 15, 30, 45, and 60 min of carburization are 41.6, 38.8, 37.4, and 35.8 emu/g, respectively. As the ratio of Ni_3_C in NICs increases, Ms decreases. Ni is a well-known ferromagnetic element, while Ni_3_C is known to be non-ferromagnetic. The gradual decrease in Ms can be explained by the magnetization reversal due to two causes: (1) The amount of Ni inside the particles is consumed due to the formation of the shell, and (2) the magnetic field transmission is disturbed due to the presence of the non-ferromagnetic shell [39,40].

## 4. Conclusions

In summary, NICs were formed by the surface modification of TEG at low temperatures (below 573 K). The Ni_3_C layer was formed by the diffusion of activated C atoms into the Ni particles on the surface. Nanoparticles with an average size of 120 nm and various ratios of Ni_3_C in NICs were produced by controlling the carburization process time. As the processing time increased, in the XRD pattern, the diffraction peak of Ni disappeared, and the diffraction peak of Ni_3_C appeared, indicating that Ni was transformed into Ni3C. The TEM analysis confirmed that the produced Ni particles were polycrystalline, and the Ni_3_C shell gradually thickened with the processing time. The change in the peak observed by XPS analysis was consistent with the XRD and TEM results, confirming that the Ni_3_C content of the reactant increased with the processing time. Thus, the growth mechanism could be elucidated. This proposed mechanism can guide the synthesis of similar core-shell nanomaterials. The magnetic properties of Ni can be controlled through the structure of the magnetic Ni core and non-magnetic Ni_3_C shell, and the oxidation resistance is expected to improve. This structure is expected to aid in the study of magnetization reversal mechanisms.

## Data Availability

Not applicable.
